# Changes in Medicaid Fee-for-Service Benefit Design for Substance Use Disorder Treatment During the Opioid Crisis, 2014 to 2021

**DOI:** 10.1001/jamahealthforum.2023.2502

**Published:** 2023-08-11

**Authors:** Angela Shoulders, Christina M. Andrews, Melissa A. Westlake, Amanda J. Abraham, Colleen M. Grogan

**Affiliations:** 1 Department of Economics, Darla Moore School of Business, University of South Carolina, Columbia; 2 Department of Health Services Policy and Management, Arnold School of Public Health, University of South Carolina, Columbia; 3 Department of Public Administration and Policy, School of Public and International Affairs, University of Georgia, Athens; 4 Center for Health Administration Studies, Crown School of Social Work, Policy, and Practice, The University of Chicago, Chicago, Illinois

## Abstract

**Importance:**

Medicaid is the largest payer of substance use disorder treatment in the US and plays a key role in responding to the opioid epidemic. However, as recently as 2017, many state Medicaid programs still did not cover the full continuum of clinically recommended care.

**Objective:**

To determine whether state Medicaid fee-for-service (FFS) programs have expanded coverage and loosened restrictions on access to substance use disorder treatment in recent years.

**Design, Setting, and Participants:**

In 2014, 2017, and 2021, a survey on coverage for substance use disorder treatment was conducted among state Medicaid programs and the District of Columbia with FFS programs. This survey was completed by Medicaid program directors or knowledgeable staff. Data analysis was performed in 2022.

**Main Outcomes and Measures:**

The following were calculated for a variety of substance use disorder treatment services (individual and group outpatient, intensive outpatient, short-term and long-term residential, recovery support, inpatient treatment and detoxification, and outpatient detoxification) and medications (methadone, oral and injectable naltrexone, and buprenorphine): (1) the percentage of Medicaid FFS programs covering these services and medications and (2) the percentage of Medicaid FFS programs using utilization management policies, such as copayments, prior authorizations, and annual maximums.

**Results:**

This study had response rates of 92% in 2014 and 2017 (47 of 51 states) and 90% in 2021 (46 of 51 states). For the 2021 wave, data are reported for the 38 non–managed care organization plan-only states. Between 2017 and 2021, coverage of individual and group outpatient treatment increased to 100% of states, and use of annual maximums for medications decreased to 3% or less (n ≤ 1). However, important gaps in coverage persisted, particularly for more intensive services: 10% of Medicaid FFS programs (n = 4) did not cover intensive outpatient treatment, 13% (n = 5) did not cover short-term residential care, and 33% (n = 13) did not cover long-term residential care. Use of utilization controls, such as copays, prior authorizations, and annual maximums, decreased but continued to be widespread.

**Conclusions and Relevance:**

In this survey study of state Medicaid FFS programs, increases in coverage and decreases in use of utilization management policies over time were observed for substance use disorder treatment and medications. However, these findings suggest that some states still lag behind and impose barriers to treatment. Future research should work to identify the long-term ramifications of these barriers for patients.

## Key Points

**Question:**

How have coverage and utilization management policies for substance use disorder treatment varied over time for Medicaid fee-for-service programs?

**Findings:**

In this survey study of state Medicaid programs conducted in 2014, 2017, and 2021, coverage for substance use disorder treatment and medications increased, whereas use of utilization management policies decreased over time. However, barriers to receiving more intensive treatment services still existed in some states.

**Meaning:**

These findings suggest that access to care for substance use disorder is improving, but restrictions on coverage persist in many states.

## Teaser

This survey study assesses changes in coverage and utilization management policies for substance use disorder treatment for Medicaid fee-for-service programs between 2014 and 2021.

## Introduction

Despite massive public investment, substance use disorder (SUD) remains an urgent public health challenge facing the US. Accidental drug poisoning has emerged as a leading cause of death for US individuals and accounts for more than one-third of unintentional, injury-related deaths.^1^ In 2021 alone, there were more than 106 000 estimated drug-related overdose deaths, the highest number of overdose deaths ever recorded in a 12-month period to date.^2,3,4,5^

Decades of research have shown that SUD, especially opioid use disorder (OUD), can often be effectively managed with a combination of medication and psychosocial intervention.^6,7^ However, ensuring access to treatment for all who need it remains a challenge. It is estimated that fewer than 20% of US individuals with SUD received any past-year treatment for SUD.^8^ Among those with OUD, which carries an especially high risk of overdose and death, receipt of any medication approved by the US Food and Drug Administration (FDA) for the treatment of OUD remains low. In 2019, only one-quarter of US individuals with OUD received any FDA-approved medication for the treatment of OUD.^9^

Although access to care is undoubtably influenced by a broad range of factors, the evidence is clear that health insurance coverage plays a crucial role.^10^ Because Medicaid is the largest payer of SUD treatment in the US and covers 38% of all individuals with OUD, it plays a key role in facilitating access to care.^11^ In 2017, Medicaid paid for OUD treatment for slightly more than half of all individuals who received it nationwide.^11^ The choices that state Medicaid programs make regarding coverage affect access to treatment for the 2 million individuals with SUD enrolled.^12^

In addition to defining SUD coverage, state Medicaid programs also employ utilization management protocols to manage access to SUD treatment. Such policies may meaningfully align use with medical necessity and/or controlling costs, which are the stated purposes of such controls. However, research suggests that utilization controls may play a role in restricting access to needed care and can prevent enrollees with SUD from initiating and remaining in treatment.^13,14,15^

Although approximately 70% of Medicaid enrollees are now serviced by managed care plans,^16^ benefits policies for state fee-for-service (FFS) programs are influential for 3 major reasons. First, state FFS programs set the minimum coverage standard for which all Medicaid managed care organization (MCO) plans in that state must comply or obtain a special waiver to provide a comparable service in lieu of a service specified in the state Medicaid plan. In other words, Medicaid MCOs are required to cover at a minimum what is specified in the state plan amendment for its FFS program.^17^ Second, as of 2020, 10 states covering more than 4.3 million individuals exclusively use FFS programs.^18,19^ Finally, 15 state Medicaid programs that contract with MCO plans carve out at least some SUD treatments to FFS programs. Given this, roughly half of states cover at least some SUD treatments through FFS. Hence, understanding coverage in Medicaid FFS programs is of critical importance.

Surveys of FFS benefits within state Medicaid programs conducted by this research team in 2014 and 2017 revealed improvements in benefits for SUD treatment.^20,21^ However, many gaps in treatment persist. It remains to be seen whether state Medicaid programs have continued to expand benefits for SUD treatment since 2017. In this study, we document how benefits have changed across the continuum of SUD treatment recommended by the American Society of Addiction Medicine (ASAM) since that time.^22^ In light of the staggering number of drug-related overdoses and deaths and Medicaid’s crucial role in providing treatment for the millions of US individuals with SUD, it is important to document state Medicaid FFS coverage and utilization control policies for SUD treatment services and OUD medications.

## Methods

### Study Design and Data

The University of Chicago Survey Lab conducted an internet-based survey of Medicaid programs in the 50 states and the District of Columbia to collect information on Medicaid FFS coverage and utilization controls for SUD treatment. The survey was conducted in 2014, 2017, and 2021. State Medicaid directors received a packet via mail or email that included details of the study, an invitation to participate, and a request to appoint a knowledgeable staff member to complete the survey. Each respondent was given clear information regarding the purpose of the survey, the intended uses of the survey data, a commitment to confidentiality, and a notification that participation in the survey was optional. To increase participation, several follow-up calls and emails were sent to directors who did not respond. In cases of incomplete or missing responses, qualified research assistants reviewed public documentation on plan requirements to try to fill in missing information. The University of Chicago Institutional Review Board approved this survey study and waived informed consent because the study was deemed not human participant research. The data were analyzed after each wave (in 2015, 2018, and 2022). This study followed the Strengthening the Reporting of Observational Studies in Epidemiology (STROBE) and applicable American Association for Public Opinion Research (AAPOR) reporting guidelines for observational and survey studies.

Nine states (Colorado, Hawaii, Idaho, Nebraska, New Jersey, New York, Pennsylvania, Tennessee, and Virginia) reported that 100% of their beneficiaries aged younger than 65 years without disability were enrolled in Medicaid managed care plans. Thus, these states were excluded from our FFS analysis for the 2021 wave.

### Measures

In each wave of the survey, information was collected on coverage for various SUD treatment services such as individual and group outpatient, intensive outpatient, short-term and long-term residential, recovery support, inpatient treatment and detoxification, and outpatient detoxification services. The survey also included data on coverage for FDA-approved medications for treating OUD, including methadone, oral and injectable naltrexone, and buprenorphine. The research team selected these measures based on key modalities for treatment services and medications based on ASAM guidelines.^23^ Because we did not collect data on alcohol use disorder medications across all 3 waves and this study focused on changes over time, we report data on OUD medications.

For each of the services and medications listed, the survey used dichotomous variables to determine whether programs had implemented the following utilization control policies: copayments, preauthorization, and annual service limits. These policies were included because they are commonly used by state Medicaid programs to regulate SUD treatment^20^ and have been the focus of substantial public debates related to behavioral health treatment parity.^24^

### Statistical Analysis

Several measures of coverage and utilization management were constructed using our data. First, we calculated the percentage of Medicaid FFS programs that offered coverage for each treatment service (individual and group outpatient, intensive outpatient, short-term and long-term residential, recovery support, inpatient treatment and detoxification, and outpatient detoxification) and medication (methadone, oral and injectable naltrexone, and buprenorphine). Next, we calculated the percentage of programs using different utilization management policies (copayments, prior authorizations, and annual maximums) for each treatment and medication. Because we were looking at the full population of Medicaid plans, we did not conduct any formal statistical tests. In cases in which 1 or more states did not answer a particular question and research assistants were unable to fill in the missing data, the number of respondents to the particular question was used as the denominator.

Data analysis was performed in 2022, using Microsoft Excel 365.

## Results

In 2014 and 2017, 47 of 51 Medicaid programs (92%) participated in our survey. In 2021, 46 of 51 Medicaid programs (90%) participated; we report data for the 38 non–MCO-only states for this wave (although due to research assistant–collected data, some variables include data for up to 40 non-MCO states). All results are provided in eTables 1 and 2 in Supplement 1. Nonrespondent states are listed in eTable 3 in Supplement 1.

### Benefit Coverage

**Figure 1.**
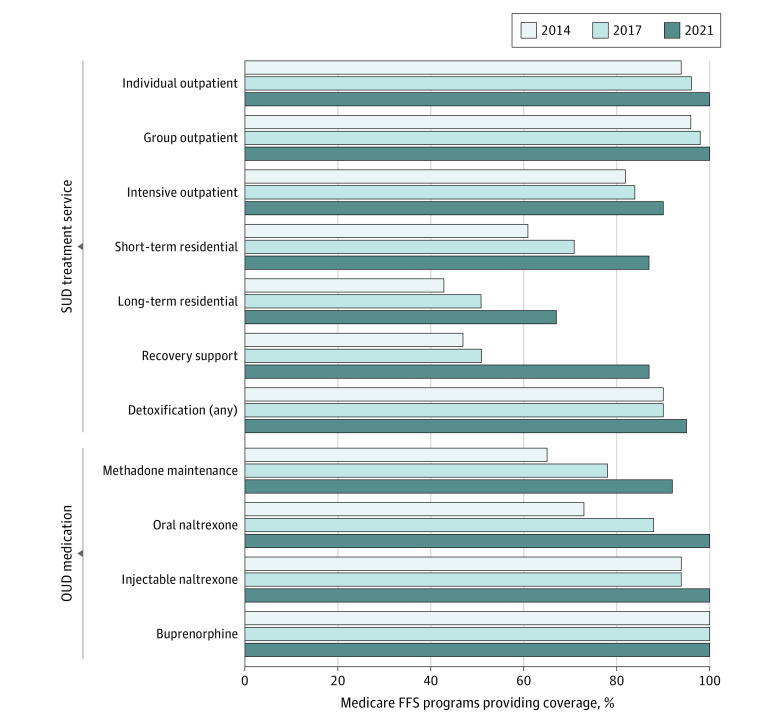
Medicaid Fee-for-Service (FFS) Programs Providing Coverage for Substance Use Disorder (SUD) Treatment Services and Opioid Use Disorder (OUD) Medications, 2014 to 2021

Coverage for all types of SUD treatment services and OUD medications increased or remained flat from 2014 to 2017 to 2021 (Figure 1). The percentage of Medicaid FFS programs that covered individual and group outpatient treatment increased to 100% in 2021 (n = 40). A few FFS programs also increased coverage for intensive outpatient treatment and detoxification (inpatient, outpatient, or both), resulting in 90% and 95% of programs (n = 36 and 38, respectively) covering these treatments, respectively. The 2021 survey was the first wave to break out inpatient and outpatient detoxification coverage separately; 67% of Medicaid FFS programs (n = 30) reported covering outpatient detoxification and 93% of programs (n = 37) reported covering inpatient detoxification. Some of the largest increases in coverage were observed for short-term and long-term residential treatment. Coverage for short-term residential programs increased from 71% in 2017 to 87% in 2021 (n = 36 and 34, respectively), and coverage for long-term residential programs increased from 51% in 2017 to 67% in 2021 (n = 26 for both). State Medicaid coverage for recovery support services also grew, increasing from 51% in 2017 to 87% in 2021 (n = 26 and 34, respectively).

Coverage for OUD medications also increased during the period from 2017 to 2021. Coverage for oral naltrexone increased from 88% of FFS programs to 100% in 2021 (n = 45 and 40, respectively); injectable naltrexone coverage also increased from 94% to 100% (n = 48 and 40, respectively). Methadone coverage increased from 78% to 92% (n = 40 to 36). Oral buprenorphine coverage remained at 100% in 2014, 2017, and 2021 (n = 51, 51, and 40, respectively).

### Copayments

**Figure 2.**
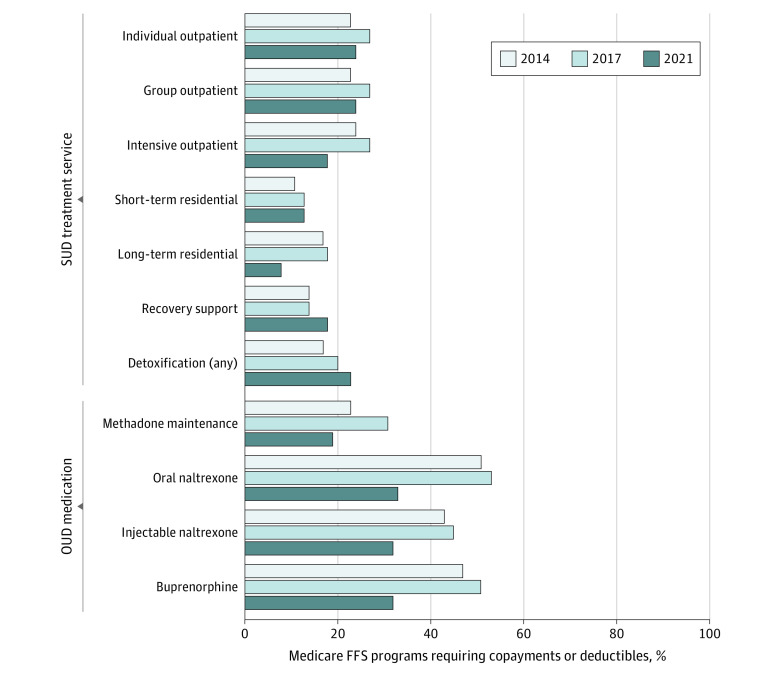
Medicaid Fee-for-Service (FFS) Programs Requiring Copayments or Deductibles for Substance Use Disorder (SUD) Treatment Services and Opioid Use Disorder (OUD) Medications, 2014 to 2021

The percentage of Medicaid FFS programs requiring copayments or deductibles increased universally between 2014 and 2017, but results were mixed in 2021 (Figure 2). Copayment requirements decreased modestly for individual and group outpatient treatment (from 27% to 24% for both services; n = 12 to 9). Copayment requirements for intensive outpatient treatment also decreased from 27% in 2017 to 18% in 2021 (n = 10 to 6). Use of copayments for short-term residential programs was stagnant at 13% (n = 4), whereas copayments decreased for long-term residential programs from 18% to 8% (n = 4 to 2). Finally, copayments increased from 14% to 18% (n = 3 to 6) for recovery support and from 20% to 23% (n = 8 to 9) for detoxification.

Copayment requirements for OUD medications universally decreased in 2021. This is a reversal from the change between 2014 and 2017: for all OUD medications, the proportion of FFS programs requiring copayments increased slightly between 2014 and 2017. The proportion of FFS programs with copayment requirements for methadone decreased from 31% in 2017 to 19% in 2021 (n = 11 to 7). The proportion of FFS programs with copayment requirements for oral naltrexone decreased the most, from 53% in 2017 to 33% in 2021 (n = 25 to 13). Programs with copayment requirements decreased for injectable naltrexone (22 [45%] to 12 [32%]) and buprenorphine (25 [51%] to 12 [32%]) between 2017 and 2021.

### Prior Authorization

**Figure 3.**
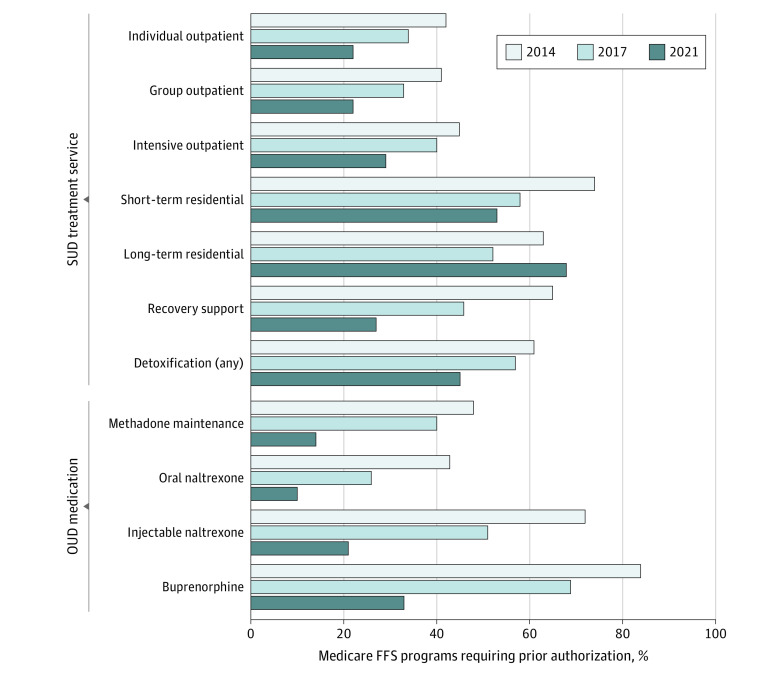
Medicaid Fee-for-Service (FFS) Programs Requiring Prior Authorization for Substance Use Disorder (SUD) Treatment Services and Opioid Use Disorder (OUD) Medications, 2014 to 2021

Prior authorization requirements for treatments and medications were highest in 2014 and decreased in both 2017 and 2021 for nearly all services (Figure 3). From 2017 to 2021, the proportion of FFS programs requiring prior authorization for individual outpatient treatment decreased from 34% to 22% (n = 15 to 8) and from 33% to 22% (n = 15 to 8) for group outpatient treatment. The proportion of programs requiring prior authorization decreased for intensive outpatient (15 [40%] to 10 [29%]), detoxification (23 [57%] to 18 [45%]), and short-term residential (18 [58%] to 17 [53%]) treatment. Long-term residential treatment was the only treatment service with an increase in the percentage of programs requiring prior authorization (12 [52%] to 17 [68%]). Recovery support services decreased most dramatically, from 46% in 2017 to 27% in 2021 (n = 10 to 9).

Prior authorization decreased precipitously for all OUD medications. For some medications, the proportion of FFS programs requiring it decreased by two-thirds. From 2017 to 2021, the proportion of programs requiring prior authorization for methadone decreased from 40% to 14% (n = 15 to 5). The proportion of programs requiring prior authorization for oral naltrexone and injectable naltrexone decreased from 26% to 10% (n = 12 to 4) and 51% to 21% (n = 25 to 8), respectively. Buprenorphine prior authorization decreased from 69% to 33% of programs (n = 35 to 13).

### Annual Service Limits

**Figure 4.**
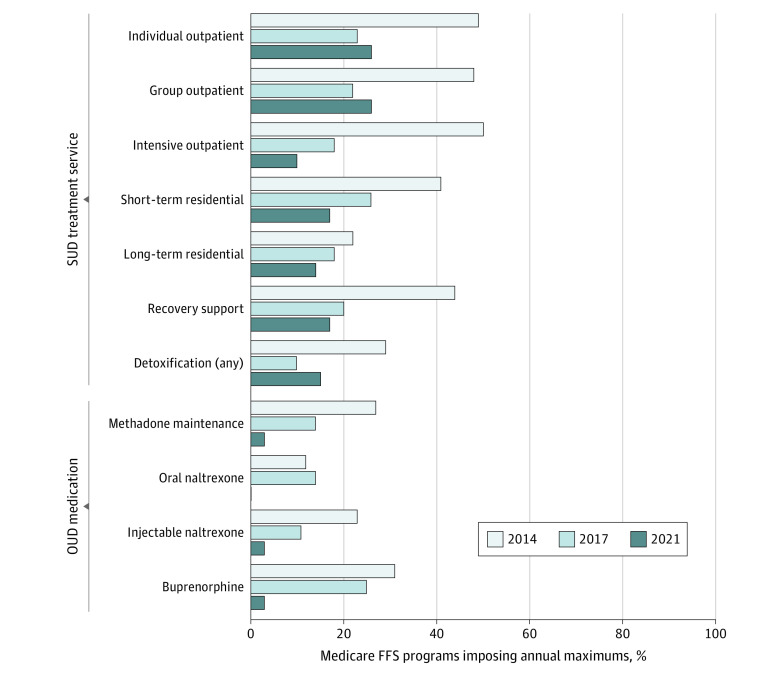
Medicaid Fee-for-Service (FFS) Programs Imposing Annual Maximums on Substance Use Disorder (SUD) Treatment Services and Opioid Use Disorder (OUD) Medications, 2014 to 2021

Annual service limits for SUD treatment peaked in 2014, decreased dramatically in 2017, and stayed relatively flat in 2021 (Figure 4). Between 2017 and 2021, there were modest increases in the percentage of FFS programs requiring annual limits for individual outpatient counseling (10 [23%] to 9 [26%]), group outpatient counseling (10 [22%] to 9 [26%]), and detoxification (4 [10%] to 6 [15%]). Annual maximums decreased for short-term residential treatment (8 [26%] to 5 [17%]), long-term residential treatment (4 [18%] to 3 [14%]), recovery support services (4 [20%] to 5 [17%]), and intensive outpatient care (7 [18%] to 3 [10%]).

From 2017 to 2021, the proportion of FFS programs requiring annual maximums for OUD medications decreased nearly to the point of nonexistence. The percentage of programs requiring this utilization control for any OUD medications decreased to 3% or less (n ≤ 1).

## Discussion

Our findings suggest that Medicaid FFS benefits for SUD treatment have continued to improve since 2017. Coverage for SUD treatment and OUD medications increased substantially over the study period. These improvements are likely driven by the continued opioid crisis, the concurrent growth in demand for affordable SUD treatment, and the mandated expansion of coverage of medications for OUD under the Substance Use-Disorder Prevention That Promotes Opioid Recovery and Treatment for Patients and Communities (SUPPORT) Act. As of January 2020, the SUPPORT Act required all Medicaid programs and plans to cover all FDA-approved medications for OUD (with some exceptions). It did not, however, prohibit the use of utilization management policies.

Overall, use of utilization controls declined for both SUD treatment services and medications. One potential mechanism for these changes is compliance with the Paul Wellstone and Pete Domenici Mental Health Parity and Addiction Equity Act (MHPAEA) of 2008, which requires insurers to eliminate inequities in coverage between SUD treatment and physical health care. An extension of parity requirements in the MHPAEA to Medicaid occurred with the passage of the Affordable Care Act in 2010, but the US Centers for Medicare & Medicaid Services (CMS) did not require full compliance until late 2017.^25^ While the extension of parity requirements to Medicaid under the MHPAEA only applied to Medicaid managed care, it also likely affected state FFS programs since, as noted above, it is still common for some states to administer behavioral health services—and in some cases, all medications—for their managed care enrollees through FFS. In particular, this may be a contributing factor for annual maximum policies dropping to nearly 0. While it is possible that some changes due to the MHPAEA were captured in the 2017 survey, the timing of compliance and our survey make it likely that some changes did not go into effect until after that wave and thus would be captured in the most recent wave. At the same time, several states partially restricted or completely banned the use of prior authorization for at least some medications for OUD, which contributes to the decreases seen in prior authorizations.^26^ Some states also placed limits on the use of copays for FDA-approved medications used to treat OUD (buprenorphine, methadone, and naltrexone).^27,28,29,30^

We observed a large increase in coverage for short- and long-term residential treatment for SUD, which had been stagnant at a low level of coverage in 2014 and 2017. The magnitude of this change may be especially important, given CMS’s option to allow states to waive the institutions for mental disease exclusion, which limited Medicaid coverage to small residential programs providing care to fewer than 16 persons. Prior to the option to waive the exclusion, states’ decision to cover residential treatment may have had a more limited impact on access to care.

Coverage for recovery support services also increased dramatically from 2014 to 2021. This may be due to the relatively low cost of providing recovery support services—making it easier for states to finance recovery support compared with other forms of care—as well as a growing body of evidence showing the importance of these services in engaging individuals with SUD in treatment and retaining them in care over time.^31,32^

While the improvements in coverage of treatment and medications are certainly encouraging, the challenge of ensuring access to the ASAM continuum of coverage for all individuals with OUD is far from resolved. Critical gaps in coverage remain. In addition to the lack of universal coverage for recovery support services, 10% of surveyed FFS programs did not cover intensive outpatient treatment, 13% did not cover any type of residential treatment, and 8% did not cover methadone. It is critical that enrollees have access to the full continuum of care, including more intensive outpatient services and residential care as well as all FDA-approved medications for the treatment of OUD.^23^ However, coverage is only one necessary component in ensuring access to care. Other factors, such as decreasing stigma and increasing the supply of SUD treatment providers and treatment facilities, are important and related to access to coverage. For example, some rural states could not comply with the SUPPORT Act mandate to cover methadone because there were no Medicaid providers who could dispense it in the state. One important step in alleviating the shortage of treatment providers may be to increase Medicaid reimbursement rates, which may increase the willingness of some existing providers to serve Medicaid clients or may induce others to enter the field altogether.

Additionally, 90% of surveyed FFS programs still impose some type of utilization management policy for SUD treatment. Especially in the midst of the opioid crisis, it is important to gain a deeper understanding of how utilization controls affect access to SUD services to help inform policy makers. Do these controls reduce unnecessary care, as intended, or do they impede access to needed care?^26,33,34^

### Limitations

This study relied on information provided by staff in each state’s Medicaid agency. Although this provides the most up-to-date information, it may be subject to unintentional reporting error. To reduce this potential for error, and to fill in missing information, we cross-checked information from public records. In cases in which respondents indicated coverage policies that were not in compliance with the SUPPORT Act, we followed up to confirm their responses given that our data collection effort occurred concurrently with implementation of key provisions. Furthermore, the survey did not ask any questions about program-specific or population-specific exemptions. Hence, it is possible that our results do not apply to every Medicaid enrollee in a given state.

Finally, the collection of our last wave of data overlaps with the COVID-19 pandemic, during which some restrictions on SUD care were loosened. This may have resulted in an underestimation of the use of some utilization management parameters.^35^

## Conclusions

The findings of this survey study of Medicaid FFS program coverage suggest that there have been substantial improvements in Medicaid benefits for SUD treatment services and OUD medications since 2014. Because 10 states only use Medicaid FFS, and state Medicaid FFS programs set the minimum standard for SUD treatment coverage in Medicaid MCO plans, this finding is highly salient. Nonetheless, there is still progress to be made before all Medicaid enrollees have adequate coverage for SUD treatment. States should continue to focus on expanding coverage across the ASAM continuum and minimize use of utilization management policies that may restrict access to care.

## Notes

Supplement 1. **eTable 1.** Percentage Data Corresponding to Figures 1 to 4 **eTable 2.** Count Data Corresponding to Figures 1 to 4 **eTable 3.** Nonrespondent US States for Each Survey Wave

Supplement 2.
Data Sharing Statement
