# Supplementary material for: Changes in Medicaid Fee-for-Service Benefit Design for Substance Use Disorder Treatment During the Opioid Crisis, 2014 to 2021
Source: JAMA Health Forum. 2023 Aug 11;4(8):e232502. doi: 10.1001/jamahealthforum.2023.2502 (PMC10422193; doi:10.1001/jamahealthforum.2023.2502)
Supplement: Supplement 1. — eTable 1. Percentage Data Corresponding to Figures 1 to 4 eTable 2. Count Data Corresponding to Figures 1 to 4 eTable 3. Nonrespondent US States for Each Survey Wave [file jamahealthforum-e232502-s001.pdf]

Supplementary Online Content

Shoulders A, Andrews CM, Westlake MA, Abraham AJ, Grogan CM. Changes in Medicaid fee-for-service benefit design for substance use disorder treatment during the opioid crisis, 2014 to 2021. *JAMA Health Forum*. 2023;4(8):e232502. doi:10.1001/jamahealthforum.2023.2502

eTable 1. Percentage Data Corresponding to Figures 1 to 4

eTable 2. Count Data Corresponding to Figures 1 to 4

eTable 3. Nonrespondent US States for Each Survey Wave

This supplementary material has been provided by the authors to give readers additional information about their work.

© 2023 Shoulders A et al. *JAMA Health Forum*.

eTable 1. Percentage Data Corresponding to Figures 1 to 4

	Coverage			Copayments			Prior Authorization			Annual Maximums		
	**2014**	**2017**	**2021**	**2014**	**2017**	**2021**	**2014**	**2017**	**2021**	**2014**	**2017**	**2021**
Individual outpatient	94%	96%	100%	23%	27%	24%	42%	34%	22%	49%	23%	26%
Group outpatient	96%	98%	100%	23%	27%	24%	41%	33%	22%	48%	22%	26%
Intensive outpatient	82%	84%	90%	24%	27%	18%	45%	40%	29%	50%	18%	10%
Short-term residential	61%	71%	87%	11%	13%	13%	74%	58%	53%	41%	26%	17%
Long-term residential	43%	51%	67%	17%	18%	8%	63%	52%	68%	22%	18%	14%
Recovery support	47%	51%	87%	14%	14%	18%	65%	46%	27%	44%	20%	17%
Detoxification (any)	90%	90%	95%	17%	20%	23%	61%	57%	45%	29%	10%	15%
Detoxification (outpatient)			67%			10%			24%			14%
Detoxification (inpatient)			93%			23%			49%			16%
Methadone maintenance	65%	78%	92%	23%	31%	19%	48%	40%	14%	27%	14%	3%
Oral naltrexone	73%	88%	100%	51%	53%	33%	43%	26%	10%	12%	14%	0%
Injectable naltrexone	94%	94%	100%	43%	45%	32%	72%	51%	21%	23%	11%	3%
Buprenorphine	100%	100%	100%	47%	51%	32%	84%	69%	33%	31%	25%	3%

© 2023 Shoulders A et al. *JAMA Health Forum*.

eTable 2. Count Data Corresponding to Figures 1 to 4

	Coverage			Copayments			Prior Authorization			Annual Maximums		
	**2014**	**2017**	**2021**	**2014**	**2017**	**2021**	**2014**	**2017**	**2021**	**2014**	**2017**	**2021**
Individual outpatient	48	49	40	10	12	9	18	15	8	21	10	9
Group outpatient	49	50	40	10	12	9	18	15	8	21	10	9
Intensive outpatient	42	43	36	9	10	6	17	15	10	19	7	3
Short-term residential	31	36	34	3	4	4	20	18	17	11	8	5
Long-term residential	22	26	26	3	4	2	12	12	17	4	4	3
Recovery support	24	26	34	3	3	6	13	10	9	8	4	5
Detoxification (any)	46	46	38	7	8	9	25	23	18	12	4	6
Detoxification (outpatient)			30			3			7			4
Detoxification (inpatient)			37			8			17			5
Methadone maintenance	33	40	36	7	11	7	15	15	5	8	5	1
Oral naltrexone	37	45	40	19	25	13	16	12	4	4	6	0
Injectable naltrexone	48	48	40	20	22	12	33	25	8	10	5	1
Buprenorphine	51	51	40	24	26	12	42	35	13	15	12	1

© 2023 Shoulders A et al. *JAMA Health Forum*.

eTable 3. Nonrespondent US States for Each Survey Wave

	Non-Respondent States
2014	Kentucky, Maryland, Nebraska, New York
2017	Minnesota, Nebraska, New Hampshire, Utah
2021	Iowa, South Dakota, Wisconsin, Arkansas, and Delaware

© 2023 Shoulders A et al. *JAMA Health Forum*.
