# Supplementary material for: Changes in Medicaid Fee-for-Service Benefit Design for Substance Use Disorder Treatment During the Opioid Crisis, 2014 to 2021
Source: JAMA Health Forum. 2023 Aug 11;4(8):e232502. doi: 10.1001/jamahealthforum.2023.2502 (PMC10422193; doi:10.1001/jamahealthforum.2023.2502)
Supplement: Supplement 2. — Data Sharing Statement [file jamahealthforum-e232502-s002.pdf]

Data Sharing Statement

Shoulders. Changes in Medicaid Fee-for-Service Benefit Design for Substance Use Disorder Treatment During the Opioid Crisis, 2014 to 2021. *JAMA Health Forum*. Published August 11, 2023. doi:10.1001/jamahealthforum.2023.2502

Data
Data available: No
